# Epigenetic inhibition of class I histone deacetylases by MS-275 attenuates diabetic skeletal muscle atrophy via Akt/ARK5–FoxO and myostatin–Smad signaling

**DOI:** 10.3389/fendo.2026.1788603

**Published:** 2026-03-09

**Authors:** Youngho Son, Hye-Eun Byeon, Sung-E Choi, Youngha Kim, Yu Jung Heo, Soon Beom Kwon, Jaemyung Choi, Seung Jin Han, Jayoung Jeon, Hae Jin Kim, Nami Lee, Kwan-Woo Lee

**Affiliations:** 1 Department of Endocrinology and Metabolism, Ajou University School of Medicine, Suwon, Republic of Korea; 2 Department of Biomedical Sciences, Graduate School of Ajou University, Suwon, Republic of Korea; 3 Institute of Medical Science, Ajou University School of Medicine, Suwon, Republic of Korea; 4 Department of Life Science, Hallym University, Chuncheon, Gangwon-do, Republic of Korea; 5 Department of Physiology, Ajou University School of Medicine, Suwon, Republic of Korea

**Keywords:** AKT/FoxO signaling, *db/db* mouse, diabetic sarcopenia, epigenetic regulation, histone deacetylase, myostatin, skeletal muscle atrophy, SMAD signaling

## Abstract

**Background:**

Sarcopenia is highly prevalent in individuals with diabetes and is associated with impaired physical function and increased mortality. Diabetes-associated skeletal muscle atrophy is driven by chronic inflammation, dysregulated anabolic–catabolic signaling, and activation of ubiquitin–proteasome–mediated protein degradation. Emerging evidence suggests that histone deacetylases (HDACs) act as epigenetic regulators of metabolic and inflammatory pathways; however, their role in diabetic sarcopenia remains incompletely understood.

**Methods:**

Male *db/db* mice were used as a model of diabetes-associated muscle atrophy and treated with MS-275 (entinostat), a selective class I HDAC inhibitor, for 4 weeks. Skeletal muscle mass and fiber cross-sectional area were assessed by magnetic resonance imaging and histological analysis. Inflammatory responses, myostatin signaling, and Akt/ARK5–FoxO–mediated catabolic pathways were evaluated using immunohistochemistry, quantitative PCR, ELISA, and western blotting.

**Results:**

MS-275 treatment significantly restored skeletal muscle mass and myofiber size in *db/db* mice. These effects were accompanied by marked reductions in macrophage infiltration, pro-inflammatory cytokine expression, and NF-κB activation. MS-275 also suppressed circulating myostatin levels and attenuated downstream Smad2/3 signaling. Furthermore, MS-275 restored Akt and ARK5 phosphorylation and promoted FoxO1/3 phosphorylation, resulting in decreased expression of the muscle-specific E3 ubiquitin ligases MuRF1 and atrogin-1.

**Conclusion:**

Our findings demonstrate that epigenetic inhibition of class I HDACs by MS-275 attenuates diabetes-associated skeletal muscle atrophy by coordinately suppressing inflammatory signaling and myostatin-driven catabolic pathways while restoring Akt/ARK5–FoxO signaling. These results suggest that class I HDACs are key epigenetic regulators of diabetic muscle wasting and that targeting their activity provides important mechanistic insights for preserving skeletal muscle mass in diabetic sarcopenia.

## 1 Introduction

Sarcopenia is a progressive and generalized skeletal muscle disorder characterized by the accelerated loss of muscle mass, strength, and physical function (1). With the rapid growth of the aging population worldwide, the burden of age-related conditions such as sarcopenia has increased substantially. Epidemiological studies estimate that sarcopenia affects approximately 10–40% of older adults, depending on geographic region and diagnostic criteria (2). Although predominantly observed in older individuals, sarcopenia can also develop during midlife and is strongly associated with a wide range of conditions, including physical inactivity (3), chronic inflammation (4), insulin resistance (5), malnutrition (6), cancer (7), chronic kidney disease (8), liver disease (9), and metabolic disorders (10).

The prevalence of sarcopenia is markedly higher in individuals with diabetes, a condition often referred to as diabetic sarcopenia, and is associated with impaired physical performance, frailty, and increased mortality (10–12). A recent meta-analysis reported a pooled sarcopenia prevalence of approximately 18% among individuals with diabetes, significantly exceeding that observed in non-diabetic populations (13). The interaction between diabetes and sarcopenia forms a vicious cycle that exacerbates glycemic dysregulation and worsens overall health outcomes (14). This bidirectional relationship is driven by shared pathophysiological mechanisms, including chronic inflammation, oxidative stress, and hormonal dysregulation, which collectively impair skeletal muscle regeneration and maintenance (15).

Consistent with these clinical observations, skeletal muscle atrophy is a well-recognized complication of diabetes and has been extensively characterized in both *in vivo* and *in vitro* experimental models. These studies demonstrate that diabetic conditions disrupt muscle protein homeostasis through dysregulation of anabolic and catabolic signaling pathways, activation of inflammatory cascades, and enhanced ubiquitin–proteasome–mediated protein degradation. For example, mesenchymal stromal cell therapy attenuated diabetes-induced muscle atrophy in *db/db* mice by suppressing the expression of muscle-specific E3 ubiquitin ligases and modulating autophagy-related signaling (16). By contrast, chronic hyperglycemia and diabetes-associated microRNA dysregulation impair muscle growth through suppression of insulin–Akt–dependent signaling, leading to increased expression of atrophy-related genes such as *MuRF1* and *atrogin-1* in skeletal muscle (17). In addition, treatment with glucagon-like peptide-1 receptor agonists reduced pro-inflammatory cytokine production, including interleukin (IL)-1β and IL-6, and protected against muscle injury in *db/db* mice, underscoring the contribution of inflammation to diabetic sarcopenia (18). Furthermore, FoxO transcription factors, acting downstream of insulin/Akt signaling, have been identified as central regulators of muscle atrophy–related gene expression in diabetic muscle models (19). Collectively, these findings implicate dysregulated anabolic–catabolic signaling, inflammatory pathways, and ubiquitin–proteasome activation as key drivers of diabetes-associated muscle atrophy.

Histone deacetylases (HDACs) have emerged as important epigenetic regulators of metabolic homeostasis and inflammatory responses in skeletal muscle. In our previous studies, we demonstrated that HDAC3 expression was increased in palmitic acid–treated C2C12 myotubes and that pharmacological inhibition of class I HDACs with MS-275 ameliorated high-fat/high-fructose–induced insulin resistance and inflammation in the skeletal muscle of *C57BL/6J* mice (20). Moreover, we observed elevated protein levels of HDAC1 and HDAC3 in skeletal muscle samples from patients with diabetes compared with healthy controls. These findings suggest a potential role for class I HDACs in the pathogenesis of diabetes-associated skeletal muscle dysfunction.

In this study, we investigated the molecular mechanisms underlying diabetic sarcopenia using a *db/db* mouse model and explored whether pharmacological inhibition of class I HDACs with MS-275 could mitigate diabetes-associated skeletal muscle atrophy.

## 2 Materials and methods

### 2.1 Animals

All of the animal experiments were approved by the Animal Ethics Committee of Ajou University (number 2023-0092). Ten-week-old male *C57BL/6J* and *db/db* mice were purchased from GEM Pharmatech (Nanjing, China). Only male mice were used in this study to exclude the potential confounding influence of estrogen and the estrous cycle on glucose metabolism and muscle mass regulation (21, 22). Animals were housed in a temperature-controlled room (22 ± 2°C) under a 12-h light/dark cycle, with ad libitum access to standard chow and water. After a 2-week acclimation period, mice were randomly assigned to three groups (n = 5 per group): control (*C57BL/6J*), diabetic muscle atrophy (*db/db*), and MS-275-treated *db/db* groups. The control and diabetic muscle atrophy groups received intraperitoneal injections of dimethyl sulfoxide (DMSO; Sigma-Aldrich, St. Louis, MO, USA), whereas the treatment group received MS-275 (entinostat, a class I histone deacetylase inhibitor; 10 mg/kg; MedChemExpress, Monmouth Junction, NJ, USA). Injections were administered three times a week for 4 weeks. At the end of the treatment period, mice were euthanized by gradual-fill carbon dioxide (CO_2_) inhalation, and blood and skeletal muscle tissues were harvested for subsequent analyses. Blood was collected by cardiac puncture, immediately centrifuged at 1000× *g* for 10 min at 4°C, and the plasma was stored at −80°C. The gastrocnemius (GA) and tibialis anterior (TA) muscles were carefully dissected, weighted, and either fixed or snap-frozen for further analyses. A schematic overview of the experimental design is shown in **Supplementary Figure 1**.

### 2.2 Magnetic resonance imaging analysis

*In vivo* MRI of mouse hindlimbs was performed using a 15.2-T MRI system equipped with Paravision 5.1 software (Bruker Biospin, Ettlingen, Germany), as previously described (23). A four-element ^1^H volume coil array and a 72-mm linear volume coil were used as the receiver and transmitter, respectively. Mice were anesthetized in an induction chamber with isoflurane (3% in O_2_) and then placed in the supine position in the scanner. Anesthesia was maintained with 1.0–1.5% isoflurane during image acquisition. Axial, mid-sagittal, and coronal scout images were acquired using rapid acquisition with relaxation enhancement (RARE) sequence. High resolution T2-weighted cross-sectional images were obtained with the following parameters: repetition time/echo time (TR/TE) = 5000/32 ms, field of view = 30 × 30 mm^2^, matrix size = 250 × 250, and slice thickness = 0.5 mm without interslice gaps. All images were analyzed using identical acquisition and quantification settings.

### 2.3 Histological analysis

Skeletal muscle tissues were fixed 4% paraformaldehyde in phosphate-buffered saline (PBS), embedded in paraffin, sectioned at 5 μm, and mounted on slides. Sections were stained with hematoxylin and eosin (H&E; Abcam, Cambridge, UK) according to standard protocols. Slides were scanned using an Aperio ScanScope CS slide scanner (Leica, Wetzlar, Germany). Muscle fiber cross-sectional area (CSA) was quantified with ImageJ software (version 1.48v; National Institutes of Health (NIH), Bethesda, MD, USA). CSA measurements were averaged per animal.

### 2.4 Immunohistochemistry

Macrophages infiltration in skeletal muscle tissues were assessed by F4/80 immunohistochemical staining. Frozen muscle sections were blocked with 5% normal bovine serum and incubated with a primary anti-mouse F4/80 antibody (Thermo Fisher Scientific, Waltham, MA, USA; CAT#14-4801-82), followed by a horseradish peroxidase (HRP)-conjugated rabbit anti-rat IgG secondary antibody. Color development was performed using 3,3’-diaminobenzidine (DAB) substrate (Vector Laboratories, CA, USA), and nuclei were counterstained with hematoxylin for 1 min. Stained sections were visualized and scanned using an Aperio ScanScope CS slide scanner.

### 2.5 Quantitative reverse transcription-polymerase chain reaction

Total RNA was extracted from GA or TA muscle using RNAiso Plus reagent (TaKaRa Bio, Shiga, Japan). Complementary DNA (cDNA) was synthesized using avian myeloblastosis virus (AMV) reverse transcriptase and random 9-mer primers with the TaKaRa RNA PCR Kit (version 3.0; TaKaRa Bio). Quantitative reverse transcription PCR was performed with SYBR Green chemistry (TaKaRa) on a TaKaRa TP-815 system. Primer sequences are listed in **Supplementary Table 1**. Relative mRNA expression levels were calculated using the comparative Ct method and normalized to mouse 36B4 mRNA expression.

### 2.6 Enzyme-linked immunosorbent assay

Plasma were analyzed for tumor necrosis factor-α (TNF-α; Invitrogen, OR, USA; Cat. 88-7324-88) and myostatin (ABclonal, MA, USA; Cat. #RK01885) using commercially available ELISA kits, according to the manufacturers’ instructions. Standard curves were generated for each assay, and samples were analyzed within the linear detection range.

### 2.7 Western blot analysis

GA muscle tissues were lysed on ice for 30 min in radioimmunoprecipitation assay (RIPA) buffer (Thermo Scientific, IL, USA; Cat. #89900) supplemented with a protease inhibitor cocktail (GenDepot, TX, USA; Cat. #P3100) and dithiothreitol (GoldBio, St. Louis, MO, USA; Cat. #DTT100). Lysates were centrifuged at 13,000 × g for 15 min at 4°C, and protein concentrations were determined using a Bradford Assay Kit (Bio-Rad, CA, USA; Cat. #1610747). Equal amounts of protein (5 μg per sample) were separated on 4-15% sodium dodecyl sulfate-polyacrylamide gel electrophoresis (SDS-PAGE) gels and transferred to polyvinylidene fluoride (PVDF) membranes. Membranes were blocked with 5% non-fat milk in Tris-buffered saline with 0.1% Tween-20 (TBST) for 1 h at room temperature and incubated overnight at 4°C with primary antibodies as listed in **Supplementary Table 2**. After washing, membranes were incubated with HRP-conjugated secondary antibodies for 1 h at room temperature. Following incubation with HRP-conjugated secondary antibodies, protein bands were detected using an enhanced chemiluminescence (ECL) substrate (Thermo Fisher Scientific inc., Waltham, MA, USA; Cat. #34580). The resulting images were analyzed by densitometry using ImageJ software (NIH, USA), and protein expression levels were normalized to the corresponding internal controls.

### 2.8 Statistical analysis

Each sample was measured in duplicate, and all experiments were performed at least three times using independent biological replicates. Data are presented as mean ± standard error of the mean (SEM). Comparisons among multiple groups were performed using one-way analysis of variance (ANOVA) followed by Bonferroni’s *post-hoc* test. Effect sizes were quantified as eta squared (η²), and a *post-hoc* power analysis was conducted to ensure the adequacy of the sample size. All analyses were performed using GraphPad Prism 10.6.0 (GraphPad Software Inc., CA, USA) with *p* < 0.05 considered significant.

## 3 Results

### 3.1 MS-275 treatment attenuates skeletal muscle atrophy in *db/db* mice

Our previous study demonstrated that HDAC3 protein expression was increased in palmitic acid–treated C2C12 myotubes and that MS-275 treatment effectively ameliorated high-fat/high-fructose (HF/HFr)–induced insulin resistance and inflammation in the skeletal muscle of *C57BL/6J* mice (20). In addition, we observed that HDAC1 and HDAC3 protein expression levels tended to be elevated in the skeletal muscle of patients with diabetes compared with healthy controls (**Supplementary Figure 2**). Despite the difference in sample sizes (n = 3 vs. 11), demographic characteristics—including age, sex, and body mass index (BMI)—were comparable between the groups (**Supplementary Table 3**). Based on these preliminary human findings, we investigated whether MS-275, a selective inhibitor of class I HDACs (HDAC1, HDAC2, and HDAC3), could ameliorate diabetes-induced muscle atrophy. *C57BL/6J* mice were used as controls, *db/db* mice as a model of diabetic muscle atrophy, and *db/db* mice treated with MS-275 as the treatment group (**Supplementary Figure 1**). To confirm the systemic metabolic effects of MS-275, intraperitoneal glucose tolerance tests were performed. MS-275 treatment significantly improved glucose tolerance compared with untreated *db/db* mice, indicating an improvement in insulin resistance (**Supplementary Figure 3**).

**Figure 1:**
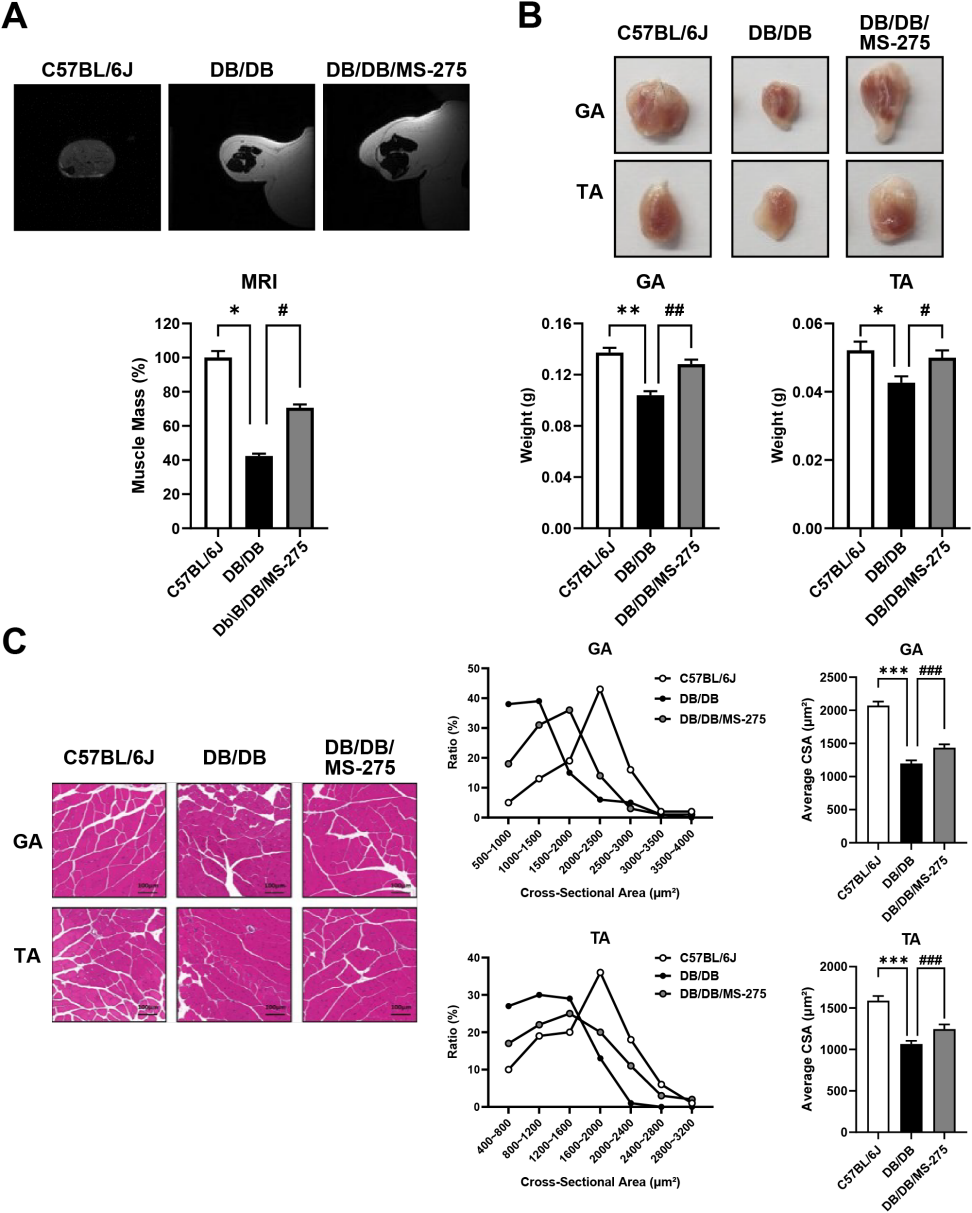
MS-275 recovers skeletal muscle atrophy in *db/db* mice. **(A)** Representative axial magnetic resonance imaging scans of hindlimb muscles from control (*C57BL/6J*), diabetic muscle atrophy (*db/db*), and MS-275–treated *db/db* mice. **(B)** Representative images and weights of gastrocnemius (GA) and tibialis anterior (TA) muscles from each group. **(C)** Hematoxylin and eosin (H&E) staining and quantification of muscle fiber cross-sectional area (CSA) in GA and TA muscles. CSAs were quantified using ImageJ software. Data are presented as mean ± SEM. Effect sizes (η²): **(A)** 0.872; **(B)** GA = 0.838, TA = 0.631; **(C)** GA = 0.912, TA = 0.781. ^*^p < 0.05, ^**^p < 0.01, ^***^p < 0.001 vs. control; ^#^*p* < 0.05, ^##^*p* < 0.01, and ^###^*p* < 0.001 vs. diabetic muscle atrophy group. SEM, standard error of the mean.

We next assessed whether MS-275 treatment also attenuated diabetes-induced muscle atrophy. MRI analysis revealed a marked reduction in skeletal muscle mass in *db/db* mice compared with control mice (**Figure 1A**). By contrast, MS-275–treated *db/db* mice exhibited a significant restoration of muscle mass. Consistent with these findings, the weights of both the gastrocnemius (GA) and tibialis anterior (TA) muscles were significantly reduced in *db/db* mice but were partially restored following MS-275 treatment (**Figure 1B**). Histological analysis using hematoxylin and eosin staining further demonstrated a significant decrease in muscle fiber cross-sectional area (CSA) in GA and TA muscles from *db/db* mice, whereas MS-275 treatment significantly increased muscle fiber CSA in both muscles (**Figure 1C**). Collectively, these results indicate that class I HDAC inhibition by MS-275 effectively attenuates diabetes-associated skeletal muscle atrophy in *db/db* mice.

### 3.2 MS-275 treatment alleviates skeletal muscle inflammation in *db/db* mice

**Figure 2:**
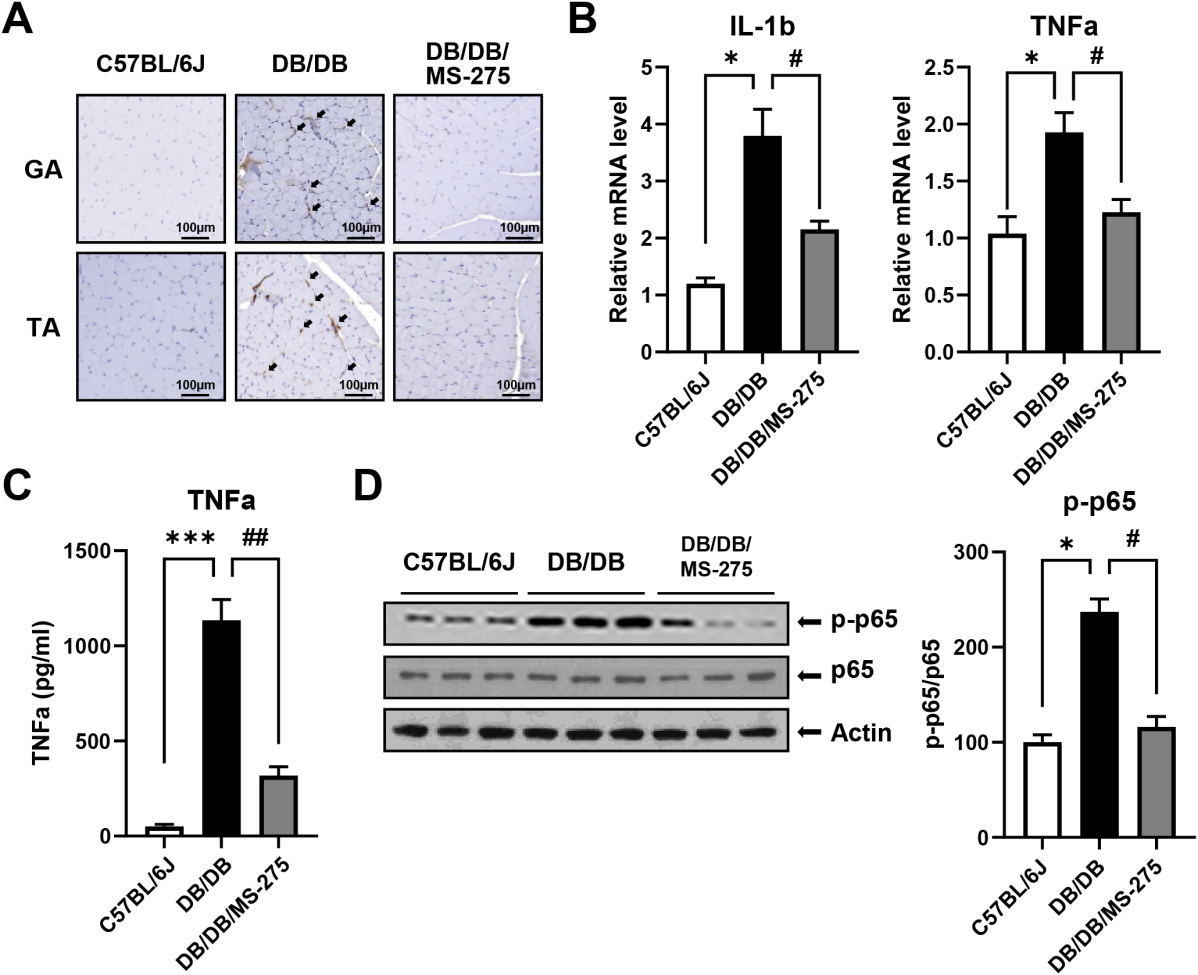
MS-275 ameliorates skeletal muscle inflammation in *db/db* mice. **(A)** F4/80 immunohistochemical staining of gastrocnemius (GA) and tibialis anterior (TA) muscles to assess macrophage infiltration in control (*C57BL/6J*), diabetic muscle atrophy (*db/db*), and MS-275–treated *db/db* mice. **(B)** mRNA expression levels of pro-inflammatory cytokines TNF-α and IL-1β in GA muscle determined by quantitative reverse transcription PCR (qRT-PCR). **(C)** Plasma TNF-α concentrations measured by enzyme-linked immunosorbent assay (ELISA). **(D)** Western blot analysis of phosphorylated p65 (p-p65) and total p65 in GA muscle. Relative protein levels were quantified using ImageJ software. Data are presented as mean ± SEM. Effect size (η²) for plasma TNF-α is 0.974. *p < 0.05, ***p < 0.001 vs. control; ^#^*p* < 0.05 and ^##^*p* <0.01 vs. diabetic muscle atrophy group. TNF-α, tumor necrosis factor alpha; IL-1β, interleukin-1 beta; SEM, standard error of the mean.

Given that skeletal muscle atrophy is frequently accompanied by chronic inflammation under diabetic conditions, we next evaluated inflammatory responses in skeletal muscle. Immunohistochemical staining for the macrophage marker F4/80 revealed markedly increased macrophage infiltration in both GA and TA muscles of *db/db* mice compared with control mice (**Figure 2A**). By contrast, MS-275 treatment significantly reduced macrophage infiltration in both muscle types. Consistent with these histological findings, mRNA expression levels of the pro-inflammatory cytokines tumor necrosis factor alpha (TNF-α) and IL-1β were significantly elevated in the GA muscle of *db/db* mice but were markedly reduced following MS-275 treatment (**Figure 2B**). In addition, circulating TNF-α levels in plasma were significantly increased in *db/db* mice and were effectively decreased by MS-275 administration (**Figure 2C**). To assess inflammatory signaling pathways further, we examined the phosphorylation status of p65. Western blot analysis showed that phosphorylated p65 (p-p65) protein levels were significantly elevated in the skeletal muscle of *db/db* mice compared with controls, indicating activation of inflammatory signaling. This increase was substantially suppressed in MS-275–treated *db/db* mice (**Figure 2D**). Together, these findings demonstrate that MS-275 treatment effectively attenuates both local and systemic inflammation associated with diabetes-induced muscle atrophy.

### 3.3 MS-275 treatment suppresses the expression of muscle atrophy–related proteins in *db/db* mice

**Figure 3:**
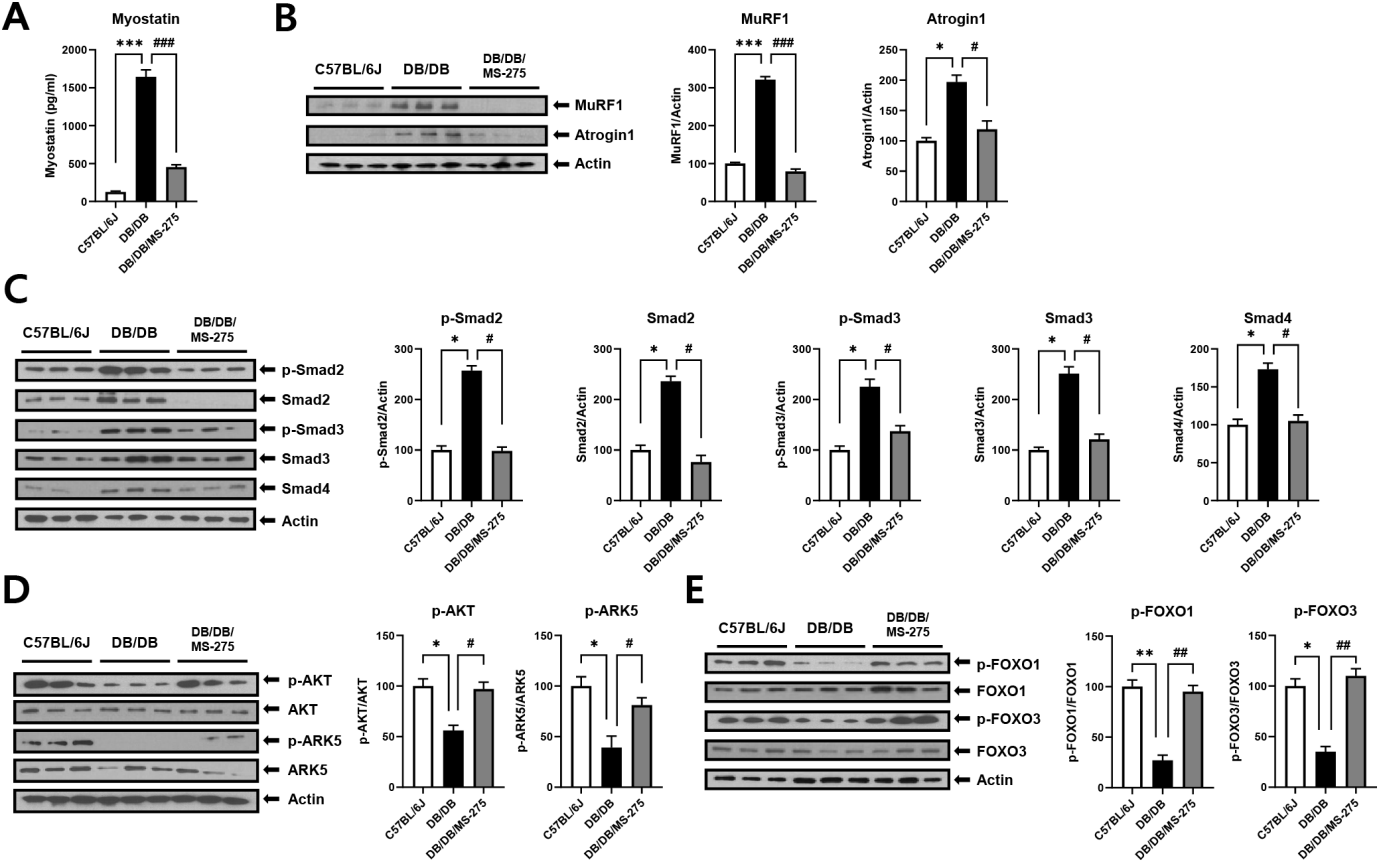
MS-275 attenuates muscle atrophy–related signaling via Smad2/3 and Akt/ARK5 pathways in *db/db* mice. **(A)** Plasma myostatin concentrations measured by enzyme-linked immunosorbent assay (ELISA) in control (*C57BL/6J*), diabetic muscle atrophy (*db/db*), and MS-275–treated *db/db* mice. **(B)** Western blot analysis of muscle-specific RING-finger protein 1 (MuRF1) and atrogin-1 in gastrocnemius (GA) muscle. **(C)** Western blot analysis of suppressor of mothers against decapentaplegic homolog (Smad) 2, Smad3, Smad4, and their phosphorylated forms. **(D)** Western blot analysis of protein kinase B (Akt) and AMP-activated protein kinase family member 5 (ARK5) phosphorylation. **(E)** Western blot analysis of Forkhead box O (FoxO) 1 and FoxO3 phosphorylation. Relative protein levels were quantified using ImageJ software. Data are presented as mean ± SEM. Effect size (η²) for plasma myostatin is 0.928. ^*^p < 0.05, ^**^p < 0.01, ^***^p < 0.001 vs. control; ^#^*p* < 0.05, ^##^*p* < 0.01, and ^###^*p* < 0.001 vs. diabetic muscle atrophy group. TNF-α, tumor necrosis factor alpha; IL-1β, interleukin-1 beta; SEM, standard error of the mean.

Muscle atrophy is driven by an imbalance between anabolic signaling and protein degradation, with myostatin and the E3 ubiquitin ligases MuRF1 and atrogin-1 playing central roles in this process (24). We examined therefore whether MS-275 treatment modulates the expression of these key atrophy-related factors. Plasma myostatin levels were significantly elevated in *db/db* mice compared with control mice, whereas MS-275 treatment markedly reduced circulating myostatin levels (**Figure 3A**). In parallel, protein expression levels of MuRF1 and atrogin-1 in skeletal muscle were significantly increased in *db/db* mice but were substantially decreased following MS-275 treatment (**Figure 3B**). These results suggest that MS-275 attenuates diabetes-associated muscle atrophy by suppressing myostatin signaling and downstream ubiquitin–proteasome–mediated protein degradation.

### 3.4 MS-275 modulates FoxO1/3 phosphorylation through Smad2/3 and Akt/ARK5 signaling in *db/db* mice

Given the observed reduction in myostatin levels following MS-275 treatment, we next examined activation of the suppressor of mothers against decapentaplegic homolog (SMAD) signaling pathway, a major downstream effector of myostatin. Western blot analysis revealed that phosphorylation of SMAD2 and SMAD3, as well as total SMAD2, SMAD3, and SMAD4 protein levels, were significantly increased in the skeletal muscle of *db/db* mice compared with controls, indicating enhanced activation of SMAD signaling (**Figure 3C**). MS-275 treatment markedly reduced phosphorylation of SMAD2 and SMAD3, along with decreases in total SMAD2, SMAD3, and SMAD4 expression.

In addition to SMAD signaling, myostatin influences muscle protein turnover through AKT-, ARK5-, and FOXO-dependent pathways. Phosphorylation of AKT was significantly reduced in *db/db* mice but was restored following MS-275 treatment (**Figure 3D**). Similarly, phosphorylation levels of FOXO1 and FOXO3, which suppress the transcription of atrophy-related genes when phosphorylated, were decreased in *db/db* mice and significantly increased in MS-275–treated mice (**Figure 3E**). These findings indicate that MS-275 modulates myostatin downstream signaling by inhibiting Smad2/3 activation while restoring Akt/ARK5–FoxO signaling, thereby attenuating FoxO-mediated expression of muscle atrophy–related genes.

## 4 Discussion

**Figure 4:**
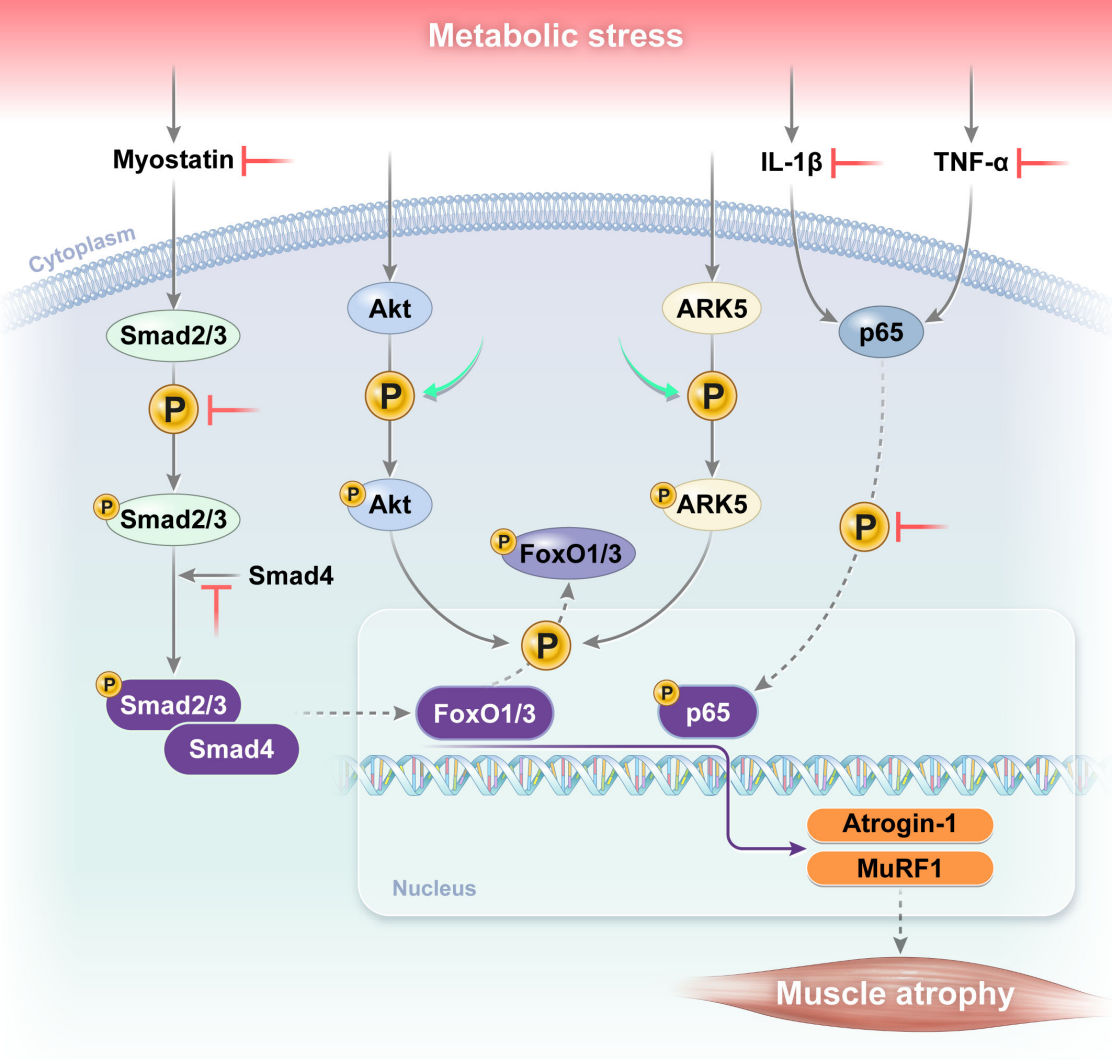
Proposed mechanisms underlying the protective effects of MS-275 against diabetic muscle atrophy. IL-1β, interleukin-1 beta; TNF-α, tumor necrosis factor alpha; Smad, suppressor of mothers against decapentaplegic homolog; Akt, protein kinase B; ARK5, AMP-activated protein kinase family member 5; FoxO, forkhead box O; MuRF1, muscle-specific RING-finger protein 1.

In this study, we demonstrate that pharmacological inhibition of class I HDACs by MS-275 effectively ameliorates diabetes-associated skeletal muscle atrophy in *db/db* mice through coordinated modulation of inflammatory signaling and myostatin-dependent catabolic pathways (**Figure 4**). MS-275 treatment restored muscle mass and myofiber CSA while suppressing inflammatory responses and atrophy-related signaling, highlighting a previously underappreciated role of class I HDACs in regulating diabetic muscle remodeling.

While these findings establish a robust molecular framework for the protective effects of MS-275, the absence of functional muscle assessments—such as grip strength or contractile force testing—is a notable limitation. Given that functional preservation is the ultimate clinical goal in sarcopenia, our data provide a mechanistic foundation for future studies that will incorporate physiological performance assays to validate the translational potential of HDAC inhibition.

Chronic low-grade inflammation is a well-established contributor to skeletal muscle wasting in diabetes, promoting proteolysis and impairing muscle regeneration through cytokine- and immune cell–mediated mechanisms. Previous *in vivo* studies have demonstrated increased macrophage infiltration and elevated pro-inflammatory cytokines, including TNF-α and IL-1β, in the skeletal muscle of diabetic models, together with activation of NF-κB signaling pathways (18, 25). Consistent with these findings, we observed marked macrophage accumulation, increased TNF-α and IL-1β expression, and enhanced p65 phosphorylation in the skeletal muscle of *db/db* mice (**Figure 2**). Importantly, MS-275 treatment significantly reduced macrophage infiltration and suppressed NF-κB activation, suggesting that class I HDAC inhibition mitigates diabetes-associated muscle inflammation. Notably, whether these anti-inflammatory effects of MS-275 represent a primary epigenetic action or a secondary consequence of improved metabolic control remains to be fully elucidated. MS-275 may directly suppress pro-inflammatory gene transcription by increasing the acetylation of NF-κB p65 or its coregulators. Simultaneously, as demonstrated by Galmozzi et al. (26), class I HDAC inhibition enhances oxidative metabolism and mitochondrial function in skeletal muscle, which may indirectly alleviate inflammation by reducing lipotoxicity and metabolic stress. Our findings likely reflect a synergistic integration of these direct and indirect mechanisms, providing a multi-layered defense against diabetes-induced muscle wasting.

Myostatin is a key negative regulator of skeletal muscle mass and is frequently upregulated under catabolic conditions such as diabetes and insulin resistance. Prior experimental studies have demonstrated that enhanced myostatin expression and activation of downstream Smad2/3 signaling contribute to muscle atrophy by inducing transcriptional programs that favor protein degradation (17). In agreement with these reports, we observed elevated circulating myostatin levels and increased activation of Smad2/3 and Smad4 in *db/db* mice (**Figures 3A, C**). Notably, MS-275 markedly suppressed myostatin expression and attenuated Smad signaling, indicating that epigenetic regulation via class I HDACs contributes to myostatin-driven muscle atrophy under diabetic conditions.

Beyond canonical Smad signaling, myostatin and inflammatory cues converge on the Akt–FoxO axis to regulate muscle-specific E3 ubiquitin ligases, including MuRF1 and atrogin-1. Reduced Akt activity leads to dephosphorylation and nuclear translocation of FoxO transcription factors, thereby promoting atrogene expression and muscle protein degradation (19). In the present study, diabetic muscle exhibited reduced phosphorylation of Akt and ARK5, accompanied by decreased phosphorylation of FoxO1 and FoxO3, consistent with enhanced FoxO transcriptional activity. MS-275 treatment restored Akt and ARK5 phosphorylation and promoted FoxO inactivation, resulting in suppression of MuRF1 and atrogin-1 expression (**Figures 3B, D, E**). Importantly, these effects occurred independently of mTOR signaling, suggesting that MS-275 primarily targets catabolic rather than anabolic pathways to preserve muscle mass under diabetic conditions. This distinction is critical, as it suggests that the attenuation of muscle atrophy is not merely a passive byproduct of improved systemic metabolism, but involves a specific epigenetic reprogramming of catabolic signaling within the skeletal muscle. While improved glucose tolerance (**Supplementary Figure 3**) likely provides a favorable systemic environment, the potent and selective suppression of atrogenes points to a direct regulatory role for class I HDACs in diabetic muscle wasting.

The observed effects of MS-275 are supported by our previous findings demonstrating that HDAC3 expression is increased under lipotoxic conditions in C2C12 myotubes and that MS-275 ameliorates insulin resistance and inflammation in skeletal muscle from high-fat/high-fructose–fed mice (20). Together with the present data, these results suggest that class I HDACs act as critical epigenetic regulators linking metabolic stress to inflammatory and myostatin-dependent signaling pathways in skeletal muscle.

While this study provides important mechanistic insights, several limitations should be acknowledged. First, as noted above, out study focused on molecular signaling without evaluating muscle strength or endurance. In addition, although MS-275 is a selective class I HDAC inhibitor, we could not delineate the isoform-specific contributions of HDAC1, HDAC2, and HDAC3 to the observed phenotypes. While our human biopsy data showed elevated HDAC1 and HAC3, we did not comprehensively assess individual HDAC isoform expression within *db/db* mouse skeletal muscle. Consequently, it remains unclear which specific isoform predominantly mediates the anti-inflammatory and anti-atrophic responses. Future research employing isoform-selective inhibitors or genetic models will be required to clarify these roles and to exclude potential non-histone effects. Furthermore, while our human skeletal muscle analysis provides potential clinical relevance, it is limited by a small, imbalanced sample size and inter-individual variability. These findings should therefore be reviewed as preliminary and hypothesis-generating. Regarding the animal model, the small sample size (n=5 per group) may limit statistical power, especially given the inherent phenotypic variability typically observed in *db/db* mice. However, to ensure the reliability and transparency of our findings, we have reported effect sizes (η²) for all primary comparisons. These values confirm that the magnitude of the observed biological changes remains substantial, supporting the validity of our mechanistic conclusions despite the inherent variability of the model. Finally, the exclusive use of male mice limits the generalizability of these findings, necessitating future investigations into potential sex-specific effects in diabetic skeletal muscle signaling.

In conclusion, our study demonstrates that epigenetic modulation by MS-275 restores skeletal muscle mass in diabetic mice by coordinately regulating Akt/ARK5–FoxO–mediated atrogene expression and myostatin–Smad signaling pathways. These findings identify class I HDACs as critical molecular nodes in diabetes-associated muscle atrophy. While further studies are required to evaluate clinical feasibility and safety, our results provide significant mechanistic insights into how epigenetic targeting of HDAC activity can influence the pathophysiological process underlying skeletal muscle wasting in diabetic conditions.

## Data Availability

The original contributions presented in the study are included in the article/**Supplementary Material.** Further inquiries can be directed to the corresponding authors.
