# Supplementary material for: Epigenetic inhibition of class I histone deacetylases by MS-275 attenuates diabetic skeletal muscle atrophy via Akt/ARK5–FoxO and myostatin–Smad signaling
Source: Front Endocrinol (Lausanne). 2026 Mar 9;17:1788603. doi: 10.3389/fendo.2026.1788603 (PMC13006271; doi:10.3389/fendo.2026.1788603)
Supplement: Supplementary file 1 [file DataSheet1.docx]

**Supplementary Material**

**Supplementary Methods**

**Study subjects and skeletal muscle biopsy**

Eleven patients with type 2 diabetes and three normoglycemic participants were enrolled in this study. Participants were categorized based on the clinical diagnosis of type 2 diabetes. Exclusion criteria included history of chronic systemic diseases (e.g., cardiovascular, hepatic, or renal failure) and use of medications known to affect skeletal muscle metabolism. Muscle biopsies were performed after an overnight fast. Percutaneous biopsy samples of the vastus lateralis muscle were obtained 15–20 cm above the knee by using a Bergström needle. Biopsy samples were immediately blotted free of blood, flash-frozen, and stored in liquid nitrogen until further analysis [1].

**Western Blot Analysis**

Western blot analysis was performed as described in the man text using primary antibodies against histone deacetylase 1 (HDAC1), HDAC2, HDAC3 (All from Cell Signaling Technology; Cat. #34589, #57156, and #85057, respectively) and anti-glyceraldehyde-3-phosphate dehydrogenase (GAPDH; Abcam, Cambridge, United Kingdom; Cat #ab9485).

**Intraperitoneal Glucose Tolerance Test (IPGTT)**

For the IPGTT, mice were fasted for 6 h, and blood samples were collected from the tail vein before peritoneal injection of 1g/kg. Additional blood samples were collected at 15, 30 and 60 min after injection. Blood glucose concentrations were measured using an Accu-Check glucometer (Roche, Mannheim, Germany).

**Supplementary Figures**

**Supplementary Figure S1. Flow chart of the animal study.** IP, intraperitoneal; IPGTT, intraperitoneal glucose tolerance test; DMSO, dimethyl sulfoxide.


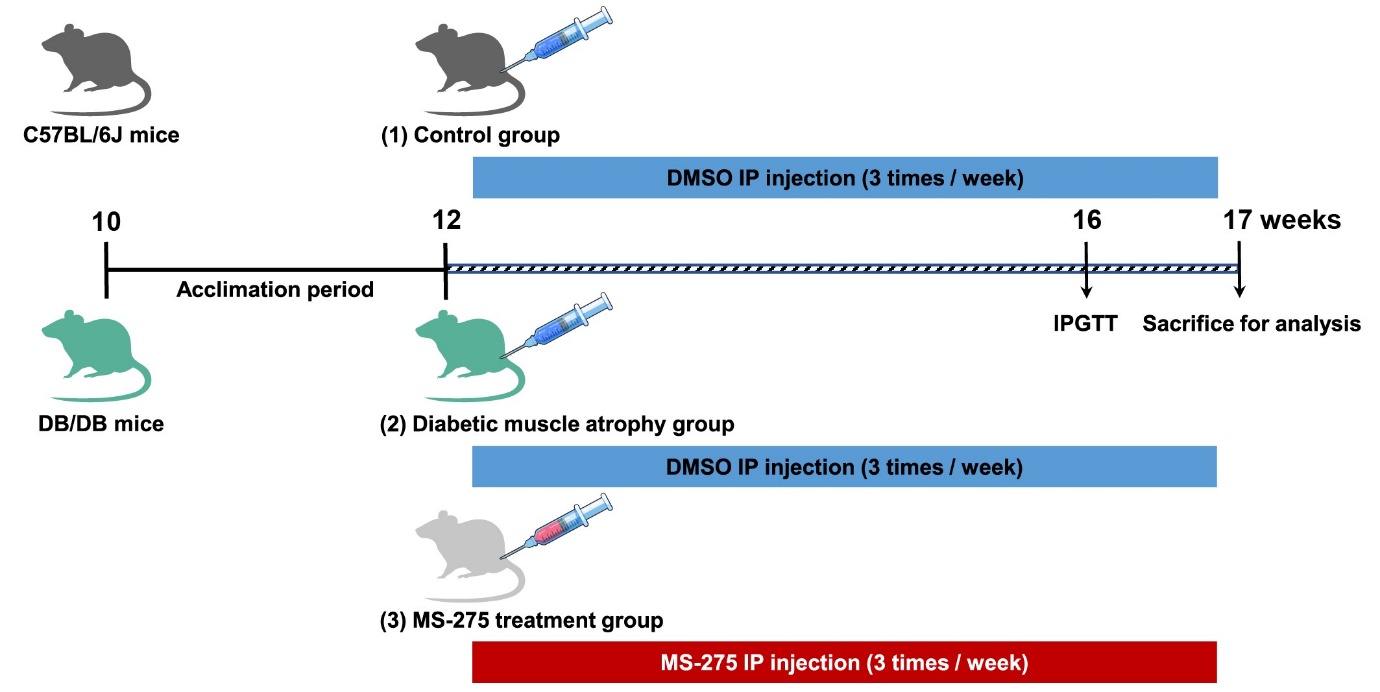


**Supplementary Figure S2. Protein levels of class I histone deacetylases (HDACs) in skeletal muscle.** To compare protein levels of class I HDACs in skeletal muscle between diabetic patients and healthy subjects, western blot analysis was performed for HDAC1, HDAC2, and HDAC3 in human skeletal muscle samples. Relative protein levels were quantified using ImageJ software. Data are presented as mean ± standard error of the mean (SEM). Statistical significance was determined using Welch’s t-test. Effect sizes (η²): HDAC1 = 0.729, HDAC2 = 0.666, HDAC3 = 0.685. ^*^*p* < 0.05 indicates significant differences compared to the healthy subjects.


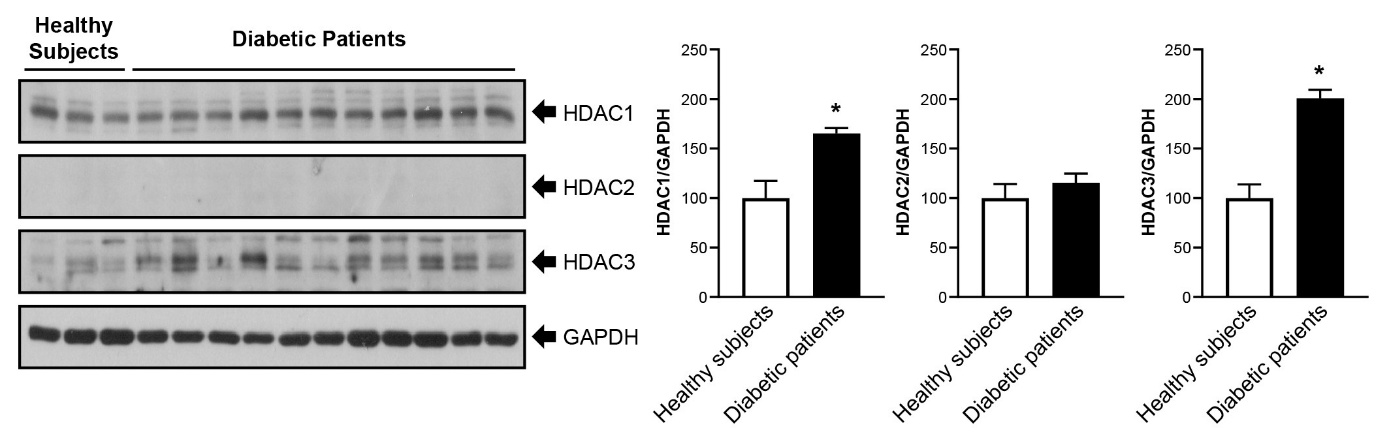


**Supplementary Figure S3. MS-275 ameliorates blood glucose levels in *db/db* mice.** Blood glucose levels were measured in the control (C57BL/6J), the diabetic muscle atrophy (DB/DB), and the MS-275 treatment (DB/DB + MS-275) groups using an intraperitoneal glucose tolerance test (IPGTT). Data are presented as mean ± standard error of the mean (SEM). ^*^*p* < 0.05, ^**^*p* < 0.01, and ^***^*p* < 0.001 indicate significant differences compared to the diabetic muscle atrophy and MS-275 treatment groups.


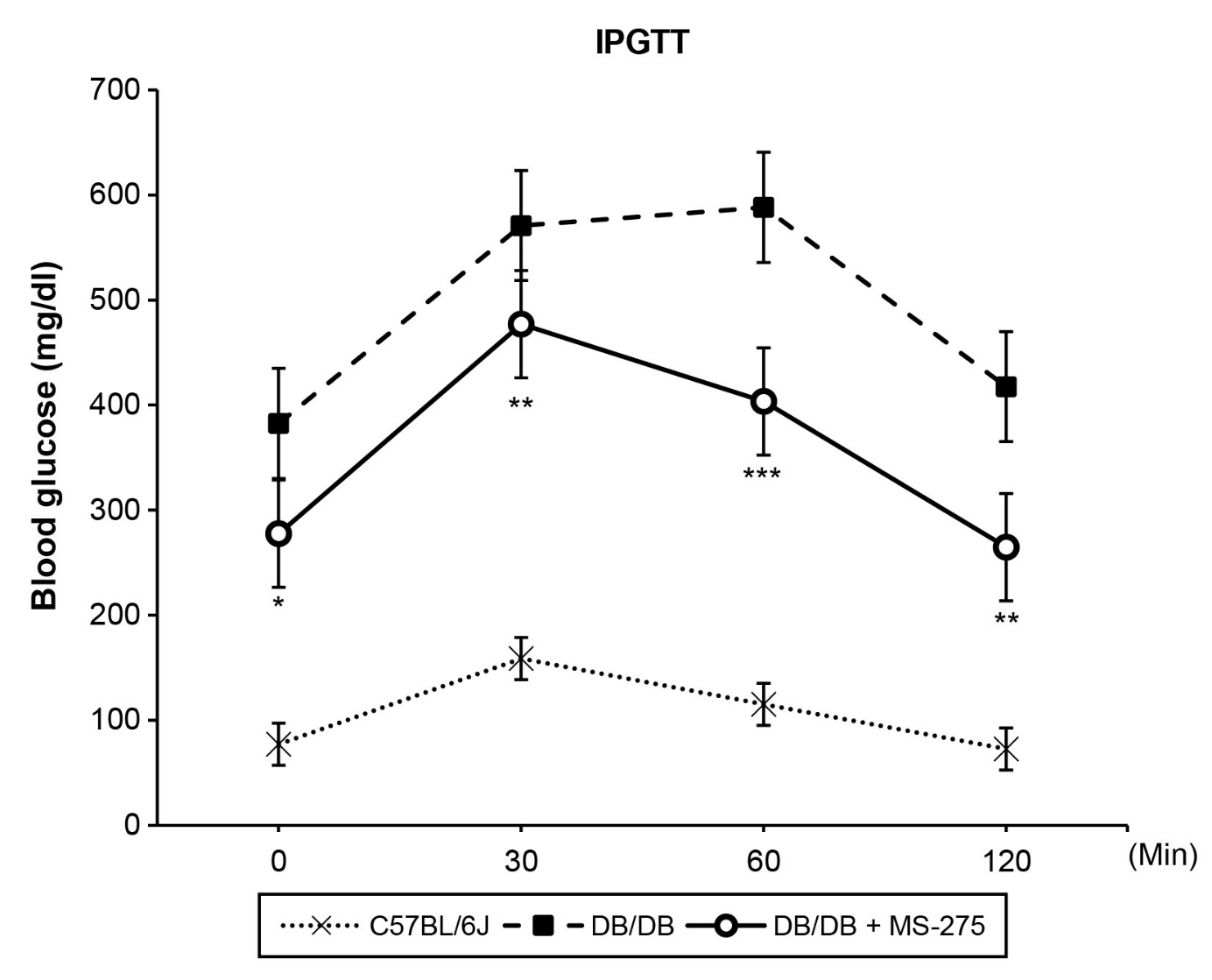


**Supplementary Tables**

Table S1. **Sequences of PCR primers**

| **Name** | **Sequence** | **Manufacturer** |
| --- | --- | --- |
| TNF-α (Forward primer) | 5 TCCAGGCGGTGCCTATGTCT 3 | Bioneer, Korea |
| TNF-α (Reverse primer) | 5 AAATCGGCTGACGGTGTGGG 3 | Bioneer, Korea |
| IL-1β (Forward primer) | 5 TCTCGCAGCAGCACATCAACA 3 | Bioneer, Korea |
| IL-1β (Reverse primer) | 5 CCTGGAAGGTCCACGGGAAA 3 | Bioneer, Korea |
| MuRF1 (Forward primer) | 5 CTTGGCACTTGAGAGGAAGG 3 | Bioneer, Korea |
| MuRF1 (Reverse primer) | 5 GAGCAGCTGGAAAAGTCCAC 3 | Bioneer, Korea |
| Atrogin-1 (Forward primer) | 5 GGCTGCTGAACAGATTCTCC 3 | Bioneer, Korea |
| Atrogin-1 (Reverse primer) | 5 AGCGCTTCTTGGATGAGAAA 3 | Bioneer, Korea |

IL-1β, Interleukin-1 beta; TNF-α, Tumor necrosis factor alpha; MuRF1, Muscle RING-finger protein-1

Table S2. **Primary antibodies used for Western blot analysis**

| **Target** | **Manufacturer** | **Catalog #** | **Host** | **Application** | **Dilution** |
| --- | --- | --- | --- | --- | --- |
| MuRF1 | ECM Biosciences | MP3401 | Rabbit | WB | 1:1,000 |
| Atrogin-1 | Abcam | ab168372 | Rabbit | WB | 1:1,000 |
| p-p65 | Cell Signaling Technology | 3033 | Rabbit | WB | 1:1,000 |
| p65 | Cell Signaling Technology | 8242 | Rabbit | WB | 1:1,000 |
| p-SMAD2 | Cell Signaling Technology | 3108 | Rabbit | WB | 1:1,000 |
| SMAD2 | Cell Signaling Technology | 5339 | Rabbit | WB | 1:1,000 |
| p-SMAD3 | Cell Signaling Technology | 9520 | Rabbit | WB | 1:1,000 |
| SMAD3 | Cell Signaling Technology | 9523 | Rabbit | WB | 1:1,000 |
| SMAD4 | Cell Signaling Technology | 38454 | Rabbit | WB | 1:1,000 |
| p-AKT | Cell Signaling Technology | 9275 | Rabbit | WB | 1:1,000 |
| AKT | Cell Signaling Technology | 9272 | Rabbit | WB | 1:1,000 |
| p-ARK5 | Invitrogen | PA5-105906 | Rabbit | WB | 1:1,000 |
| ARK5 | Cell Signaling Technology | 4458 | Rabbit | WB | 1:1,000 |
| p-FOXO1 | Cell Signaling Technology | 9461 | Rabbit | WB | 1:1,000 |
| FOXO1 | Cell Signaling Technology | 9454 | Rabbit | WB | 1:1,000 |
| p-FOXO3 | Cell Signaling Technology | 9465 | Rabbit | WB | 1:1,000 |
| FOXO3 | Cell Signaling Technology | 12829 | Rabbit | WB | 1:1,000 |
| β-actin | Santa Cruz | sc-47778 | Mouse | WB | 1:5,000 |

p-, phospho-; MuRF1, Muscle-specific RING-finger protein 1; SMAD, suppressor of mothers against decapentaplegic homolog; AKT, protein kinase B; ARK5, AMP-activated protein kinase family member 5; FOXO, forkhead box protein O

Table S3. **Characteristics of human subjects used for analysis of skeletal muscle class I histone deacetylases (HDACs)**

| **Demographics** | **Healthy subjects (N=3)** | **Diabetic patients (N=11)** | ***P* value** |
| --- | --- | --- | --- |
| Sex (Female) | 3 | 10 | - |
| Age (years) | 40.0 ± 4.4 | 44.8 ± 1.7 | 0.295 |
| BMI (kg/m^2^) | 22.9 ± 1.39 | 24.9 ± 1.5 | 0.546 |

BMI, body mass index

**References**

[1] Cui R, Choi SE, Kim TH, Lee HJ, Lee SJ, Kang Y, et al. Iron overload by transferrin receptor protein 1 regulation plays an important role in palmitate-induced insulin resistance in human skeletal muscle cells. *FASEB J.* (2019) 33:1771–86. doi: 10.1096/fj.201800448RR
